# Heterotopic Ossification of the Indirect Head of the Rectus Femoris

**DOI:** 10.5334/jbsr.3750

**Published:** 2024-11-07

**Authors:** Rob Vernelen, Filip M. Vanhoenacker

**Affiliations:** 1 KU Leuven, Belgium; 2 AZ Sint‑Maarten Mechelen and University (Hospital) Antwerp/Ghent, Belgium

**Keywords:** Heterotopic ossification, rectus femoris, indirect head, radiography, CT, MRI

## Abstract

*Teaching point:* The clues to the correct diagnosis of heterotopic ossification of the indirect head of the rectus femoris are its location above the acetabular rim and its (inverted) comma‑like morphology.

## Case History

A 52‑year‑old male presented with chronic, exercise‑induced, and load‑dependent right hip pain. No history of trauma or relevant medical or familial history was reported. Physical examination revealed a positive flexion adduction internal rotation (FADIR) sign.

**Figure 1 A‑B.**
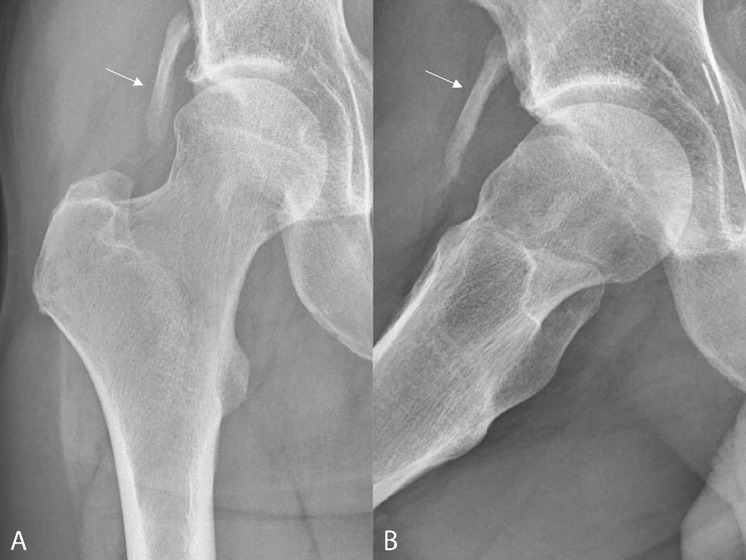
Conventional radiography shows an elongated, well circumscribed heterotopic ossification projecting superolaterally to the anterior inferior iliac spine (AIIS) (arrows). There is also cam deformity with loss of concavity of the femoral head‑neck junction.

Conventional radiography (CR) (Figure 1A,B) revealed an elongated, well circumscribed heterotopic ossification (HO) projecting superolaterally to the anterior inferior iliac spine (AIIS) (arrow). Notably there was also subtle cam deformity with loss of concavity of the femoral head‑neck junction.

**Figure 2:**
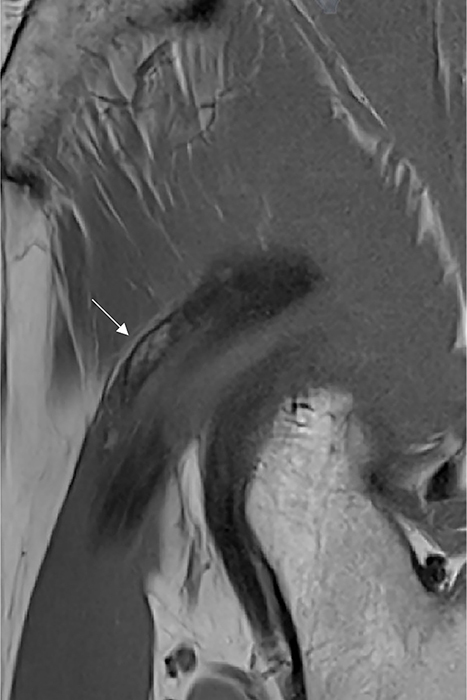
Magnetic Resonance Imaging shows mature HO containing fatty bone marrow along the course of the indirect head of the rectus femoris (arrow) on sagittal T1‑weighted images.

Subsequent magnetic resonance imaging (MRI) revealed mature HO containing fatty bone marrow along the course of the indirect head of the rectus femoris on sagittal T1‑weighted images (Figure 2, arrow).

**Figure 3 A‑B.**
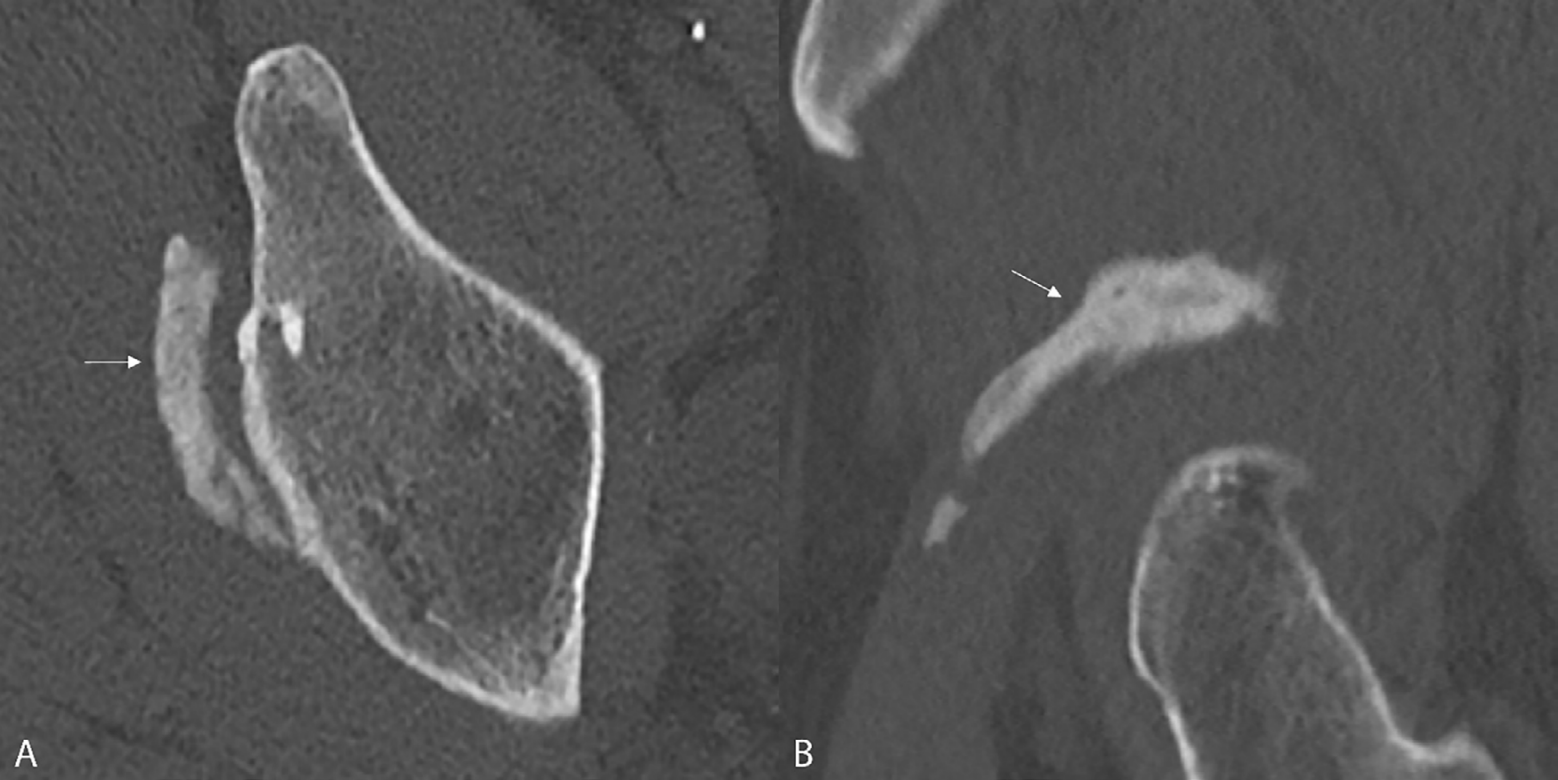
Computed Tomography confirms HO with elongated morphology superior to the acetabular rim (arrows).

Computed tomography (CT) confirmed HO with elongated morphology superior to the acetabular rim (Figure 3A,B; axial and sagittal reformatted image, arrows).

## Comments

The rectus femoris muscle has two parts: the direct head originating from the AIIS and the indirect head or reflected head with its origin superiorly to the acetabular rim.

HO consists of formation of lamellar bone in an abnormal location. HO typically follows three phases along with the progressive calcification and ossification, described as early, intermediate, and mature phases.

Although occurrence around the hip joint is common, the indirect head of the rectus femoris is rarely involved.

In most cases, a history of trauma or surgery is present, but sometimes the patient cannot clearly recall a single trauma. Potentially, these cases may be attributed to repetitive microtrauma or may be idiopathic [1]. In our case, the lesion likely resulted from an old avulsion injury during his youth while being a soccer player.

HO in the hip is often asymptomatic. In symptomatic patients, it is hypothesized that HO along the reflected head may impinge on the femoral neck during hip motion, which may contribute to the symptoms and a positive FADIR test.

CR is the mainstay in detecting HO. CT determines its precise size, extension, and the stage of the disease. MRI is useful to evaluate potential impingement of adjacent soft tissue structures.

Asymptomatic HO typically does not require treatment, while treatment of symptomatic cases consists of immobilization for immature bone and physical therapy for mature bone. Arthroscopic resection is preferred for patients with restricted motion [1].

The keys to diagnosing HO in the indirect head is identifying the lesion’s location near the posterosuperior rim of the acetabulum and its elongated morphology resembling an (inverted) comma shape along the indirect head of the rectus femoris tendon.
